# Update on a Pharmacokinetic-Centric Alternative Tier II Program for MMT—Part I: Program Implementation and Lessons Learned

**DOI:** 10.1155/2012/946742

**Published:** 2012-03-27

**Authors:** David C. Dorman, Melvin E. Andersen, Jerry M. Roper, Michael D. Taylor

**Affiliations:** ^1^College of Veterinary Medicine, North Carolina State University, Raleigh, NC 27607, USA; ^2^Institute for Chemical Safety Sciences, The Hamner Institutes for Health Sciences, Research Triangle Park, NC 27709, USA; ^3^Health, Safety, Environment, and Security, Afton Chemical Corp., Richmond, VA 23219, USA

## Abstract

Concerns have been raised regarding environmental manganese exposure since high exposures have been associated with neurological disorders. The USA Environmental Protection Agency most recent human health risk assessment of inhaled manganese conducted in 1993 identified specific areas of uncertainty regarding manganese pharmacokinetics. This led to the development of a test rule under the USA Clean Air Act that required the generation of pharmacokinetic information on the inorganic manganese combustion products of the organometallic fuel additive methylcyclopentadienyl manganese tricarbonyl (MMT). The Alternative Tier 2 testing program for MMT, described in this paper, has yielded substantial pharmacokinetic data and has enabled the generation of physiologically based pharmacokinetic (PBPK) models for manganese. These models are capable of predicting tissue manganese concentrations across a variety of dose routes, levels, and durations while accounting for factors such as age, gender, and reproductive status, enabling the consideration of tissue dosimetry in future risk assessments.

## 1. Introduction

Methylcyclopentadienyl manganese tricarbonyl (MMT^®^, a registered trademark of Afton Chemical Corporation) is an organometallic fuel additive that was developed by the Ethyl Corporation in the 1950s. MMT is currently marketed globally by Afton Chemical Corporation among others. MMT has been used in a variety of fuels, including leaded and unleaded gasoline, diesel and turbine fuel, and fuel oil to raise octane and improve combustion [1]. Manganese concentrations in unleaded gasoline typically range from 5 to 20 ppm when MMT is used. In the United States, MMT is approved for use up to 8.3 ppm in conventional unleaded gasoline.

As a fuel additive, MMT falls under the regulatory domain of the United States Environmental Protection Agency (USEPA), and MMT manufacturers and/or importers are subject to relevant provisions of the USA Clean Air Act (CAA). This paper provides an overview of the novel CAA Alternative Tier 2 test program for MMT designed to collect critical manganese pharmacokinetic data in animals (all test reports and correspondence related to the Alternative Tier 2 Testing for MMT can be found in the Federal Docket Management System (FDMS) at http://www.regulations.gov identified by docket number EPA-HQ-OAR-2004-0074.). Follow-up efforts led to the development of a series of physiologically based pharmacokinetic (PBPK) models for manganese. This paper will briefly examine the toxicology of manganese, the regulatory history of MMT, the design and conduct of the Alternative Tier 2 program for MMT, key research findings derived from the health effects research program, and critical lessons learned that can be applied to other chemicals. The second paper in this two-part series [2] describes the PBPK models in greater detail and provides a framework for their application to the risk assessment of manganese.

## 2. Manganese Toxicology: Public Health Concerns about MMT

Manganese is an essential trace metal that is required for normal amino acid, lipid, protein, and carbohydrate metabolism. Under certain high-dose exposure conditions or disease states (e.g., hepatobiliary dysfunction), however, manganese can induce adverse neurological, reproductive, and respiratory effects in humans [3]. Manganese toxicity is dependent on the dose to target tissue and develops after inhalation, oral, and parenteral exposure; however, this paper will focus predominantly on inhalation. Atmospheric sources of manganese include manmade and natural sources including wind erosion of dusts and soils. Industries associated with manganese emissions include ferroalloy production, iron and steel foundries, and power plant and coke oven combustion emissions. Ambient (background) levels of manganese in rural and urban air range from 0.005 to 0.07 *μ*g Mn/m^3^ [4].

Neurological effects occur at lower dose levels than other adverse effects, so consideration of these effects drives the human health risk assessment of inhaled manganese [4–6]. The earliest manifestations of manganese neurotoxicity (manganism) include fatigue, headache, muscle cramps, loss of appetite, apathy, insomnia, and diminished libido. As manganese exposure continues and the disease progresses, patients may develop dystonia, bradykinesia, rigidity, gait disorders, postural instability, micrographia, and muscle tremors (for review see [7]). These signs are associated with primary involvement of the globus pallidus. Individuals with chronic manganese neurotoxicity resemble patients with Parkinson's disease; however, these syndromes can be distinguished both clinically and with neuroimaging studies [7]. Although these syndromes are clinically distinct, some studies suggest that manganese overexposure may pose a risk factor for Parkinson's disease [7, 8].

A variety of inhalation exposure scenarios exist for manganese from occupational settings with mid- to high-dose exposures to much lower exposures found in the general environment. Forms of manganese include but are not limited to manganese dioxide (MnO_2_) and other oxides, manganese sulfate (MnSO_4_), manganese phosphates (MnPO_4_), and organometallic manganese compounds. Absent underlying hepatobiliary disease, frank manganese neurotoxicity has been observed in workers that have been chronically exposed to dusts or fumes that contain high levels (>1 mg Mn/m^3^) of manganese [4]. More subtle neurobehavioral effects have been reported in welders and other workers at lower (~0.2 mg Mn/m^3^) exposure concentrations [9]. One of the more influential occupational studies was performed by Roels and coworkers [10]. This cross-sectional study of male workers was used by the USEPA as their critical study for deriving their chronic inhalation reference concentration for manganese (RfC) of 0.05 *μ*g/m^3^ that was last updated in 1993 [6]. According to USEPA, an RfC is an estimate (with uncertainty spanning perhaps an order of magnitude) of a continuous inhalation exposure to the human population (including sensitive subgroups) that is likely to be without an appreciable risk of deleterious effects during a lifetime. Roels et al. [10] assessed employees from a Belgian alkaline battery production plant and controls from a polymer processing plant. Personal air samplers in the battery plant indicated an 8 hr time weighted average (TWA) exposure of 0.215 mg/m^3^ for respirable manganese. An average cumulative exposure of 1.2 mg/m^3^ *·*years of respirable manganese was associated with decrements in some neurofunctional performances (e.g., mean reaction times, eye-hand coordination and muscles tremor scores) between the exposed group and the control group. Health Canada current risk assessment relied on a study of Italian ferroalloy workers performed by Lucchini and coworkers [11] for the derivation of their RfC for manganese [5]. Lucchini examined several cohorts of workers (furnace, casting, and welding job functions) between 1981 and 1997. Manganese exposures for all classes of workers dropped appreciably during this time. For example, the geometric mean manganese concentrations (in total dust) went from 167 to 54.7 *μ*g/m^3^ in the maintenance area where welding was performed. An exposure concentration of approximately 71 *μ*g Mn/m^3^ was derived from an estimated cumulative exposure index. Lucchini reported an association between occupational manganese exposure and some neurological effects including higher symptom reporting, increased tremor frequency, altered motor function, and impaired memory. Bailey and colleagues [12] published an alternative RfC value of 2–7 *μ*g Mn/m^3^ that was based upon their use of epidemiological studies published after 1992. The American Conference of Industrial Hygienists (ACGIH) threshold limit value (TLV) for elemental manganese and related inorganic compounds is 0.2 mg Mn/m^3^ [13]. This standard is set as a time-weighted average for an 8 hr shift 5 days per week designed to protect workers in occupational settings.

Ambient manganese exposure data are needed to assess the potential public health impacts of MMT in fuel. Several of the most significant studies were performed in Canadian cities when MMT was widely used in the local fuel supply. For example, an extremely large effort using probabilistic sampling techniques measured manganese exposure levels in Toronto residents when all fuel contained MMT [14]. The results of this study and related analyses of data from personal samplers [15, 16] suggest that the median annualized nonoccupational exposure concentration was 0.008 *μ*g/m^3^ with the highest long-term exposures at or near 0.022 *μ*g/m^3^. as a respirable fraction (PM_2.5_). Roadway measurement of soil manganese concentrations did not reveal a measurable increase in manganese levels along urban Toronto highways [17]. Studies from Australia have shed additional light on the impact of MMT on atmospheric manganese concentrations [18, 19]. Gulson and coworkers reported no significant changes in environmental samples or blood manganese concentrations in children following the introduction of MMT in Sydney.

Concerns regarding the use of MMT as a gasoline fuel additive have also been influenced by the USA experience of tetraethyl lead in gasoline. Concerns about automotive emissions of lead prompted the USEPA in 1973 to phase out the use of lead in gasoline [20]. The experience with tetraethyl lead created an environment of distrust between public health officials, environmental legislators, advocacy groups, and fuel manufacturers. USEPA health effects testing program for MMT emerged from this challenging climate.

## 3. Overview of the Regulatory History of MMT

Manganese is listed as an air toxic by the USEPA. When the USEPA ordered the phasing out of leaded gasoline, MMT and other alternative octane enhancers were used in unleaded gasoline. In 1977, a Congressional amendment to the CAA banned the use of all fuel additives not “substantially similar” to gasoline, including MMT, unless the USEPA granted a waiver [21]. The prohibition on the use of MMT in gasoline was largely based on concerns that MMT use could affect the first generation of automotive emissions-control systems. Ethyl Corporation first applied for this waiver in 1978. This and several subsequent waiver petitions submitted by Ethyl Corporation were denied because of USEPA concerns regarding potential increases in exhaust hydrocarbon emissions resulting from MMT use [21].

As part of a later waiver application for MMT, the USEPA conducted a risk assessment of exposure to inhaled manganese in 1993, with the publication of an inhalation RfC of 0.05 *μ*g/m^3^ [6]. The USEPA used standard RfC development methodologies for a noncancer endpoint and based the RfC on the Roels et al. study [10] that evaluated neurobehavioral and motor movement impairments observed in workers exposed to manganese dioxide (MnO_2_). The exposure concentration reported by Roels was considered by the USEPA to be a lowest-observable-adverse-effect level (LOAEL). The LOAEL value was adjusted for continuous exposure durations, and several uncertainty factors were applied. These uncertainty factors included use of a LOAEL instead of a no-observable-adverse-effect level (NOAEL), extrapolation from subchronic to chronic, protection of potential sensitive members of the human population, and a factor reflecting other uncertainties in the database, such as less-than chronic periods of exposure, inadequate information regarding developmental and reproductive toxicity, and uncertainty about the toxicity of various forms of manganese [22]. Although the nutritional essentiality of manganese in the diet was discussed in the documentation of the RfC, it played no practical role in the calculation of an RfC [21].

The USEPA also published a risk characterization that included exposure and dose-response assessments for MMT in gasoline [23, 24]. After completing this evaluation, EPA determined that use of MMT would not “cause or contribute” to the failure of vehicle emission control systems. EPA was unable to determine, however, whether a risk to the public health occurred from use in gasoline [25]. The agency stated “[a]lthough it is not possible based on the present information to conclude whether specific adverse health effects will be associated with manganese exposures in the vicinity of or exceeding the (estimated safe level over a lifetime of exposure), neither is it possible to conclude that adverse health effects will not be associated with such exposures…Given the information that is available at present and the uncertainties discussed here, a reasonable basis exists for concern regarding potential public health risks, especially for sensitive subpopulations, if MMT were to be widely used in unleaded gasoline [25].” The USEPA also concluded that long-term animal testing and exposure research were needed to more accurately define the risk. Coincidental to these activities, in July 1994, the USEPA Administrator denied Ethyl Corporation newest waiver petition specifically because of concerns about potential risks to public health [24] and refused to register MMT for use in the USA In 1995, the Ethyl Corporation successfully challenged the denial of its petition based on public health concerns (*Ethyl Corporation v. Browner,* 51 F.3d 1053 (D.C. Cir. 1995)), as well as USEPA decision not to register MMT (*Ethyl Corporation v. Browner,* 67 F.3d 941 (D.C. Cir. 1995)). As a result, the USEPA formally approved the use of MMT in conventional unleaded gasoline and registered it as a fuel additive under the CAA, allowing for its domestic sale.

Provisions of the CAA provide the USEPA with the authority to require testing of fuels and fuel additives used in motor vehicles, including MMT, to help fill data gaps and provide information that potentially would result in a more definitive risk evaluation. These health testing requirements are addressed in Sections  211(b)(2) and 211(e) of the CAA. Section  211(b)(2) states “For the purpose of registration of fuels and fuel additives, the Administrator shall, on a regular basis, require the manufacturer of any fuel or fuel additive” to conduct “Tier 2” tests to determine potential public health and environmental effects of the fuel or additive (including carcinogenic, teratogenic, or mutagenic effects). These studies would be conducted using test procedures and protocols established by the USEPA. Moreover, the CAA also provides USEPA with the discretion to modify the standard Tier 2 health effects testing requirements for a fuel or fuel additive by substituting, adding, or deleting testing requirements or changing the underlying vehicle/engine specifications (40 CFR 79.58(c)). Health effects testing for MMT fell under this so-called “Alternative Tier 2” requirement, as the concern and subsequent testing was focused around the inorganic exhaust products of MMT and not MMT itself.

## 4. Establishment of the Alternative Tier 2 Testing Program for MMT

In 2001, the Ethyl Corporation was notified by the USEPA of the Alternative Tier 2 provisions for MMT which fell within two general categories: pharmacokinetic testing of manganese compounds and characterization of manganese emissions from vehicles utilizing fuels containing MMT. A central objective of the MMT Alternative Tier 2 program was to generate data to support development of physiologically based pharmacokinetic (PBPK) models for manganese. As a result, PBPK model development became the subject of a series of studies funded by Afton Chemical Corp. and handled in a way similar to other facets of the Alternative Tier 2 program.

**Figure 1:**
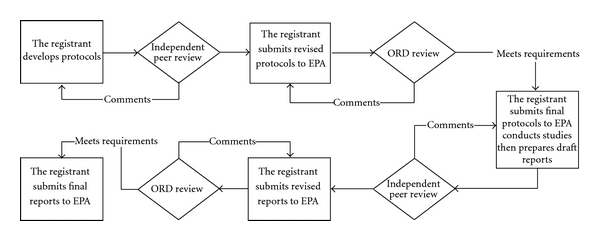
Schematic overview of steps used to develop and review study protocols and produce final reports. Independent peer review was carried out by the appropriate TAP. Completed final reports for all of the manganese studies can be found in the Federal Docket Management System (FDMS) at http://www.regulations.gov identified by docket number EPA-HQ-OAR-2004-0074. EPA: Environmental Protection Agency; ORD: EPA Office of Research and Development.

Multiple pharmacokinetic studies were performed in response to the USEPA mandate (Table 1). Studies that were required by the USEPA were performed in accordance with the USEPA Good Laboratory Practice (GLP) Standards for Inhalation Exposure Health Effects Testing (40 CFR Part 79.60). All studies were performed at The Hamner Institutes for Health Sciences (Hamner) (formerly the Chemical Industry Institute of Toxicology (CIIT)). All required study protocols, protocol amendments, and draft final reports underwent independent scientific review by project specific “technical advisory panels” (TAPs) composed of individuals with expertise in inhalation toxicology, pharmacokinetics, and neurotoxicology (Figure 1). Members of the TAPs, which changed for the different facets of the test program, were chosen by the study sponsor with input from the testing laboratory and approved by the USEPA. All study results underwent additional independent peer review during subsequent publication of the work in scientific journals.

Several critical decisions were reached early in the MMT test program. These concerned the form of manganese to be examined, animal species used, and endpoints of interest. The main focus of the test program was to evaluate combustion products of MMT. This decision was influenced in part by the observation that MMT undergoes rapid photolysis when exposed to light, forming methylcyclopentadiene, cyclopentadiene, carbon monoxide, and a manganese carbonyl that is readily oxidized to trimanganese tetroxide [26]. One early dilemma facing study toxicologists was determination of these combustion products. Early studies performed by Ter Haar and coworkers [27] showed that the primary combustion product from MMT was Mn_3_O_4_. This study used fuel spiked with large concentrations of MMT, an automobile without a catalytic converter, and X-ray diffraction methods for chemical speciation, resulting in an artifactual apparent enrichment of Mn_3_O_4_ in the exhaust. Zayed et al. [28], using scanning electron microscopy coupled with energy dispersive X-ray spectrometry, also suggested that the chemical form of emitted manganese was as an oxide but could not rule out sulfates and other manganese species. Other studies, using lower treat rates representative of actual use patterns and more advanced analytical methods, including X-ray absorption near-edge structure (XANES) spectra and K-edge X-ray absorption fine structure (XAFS) spectroscopy, showed the presence of three major manganese-containing components in tailpipe emissions: a divalent manganese phosphate, sulfate, and oxides (most likely as Mn_3_O_4_) [29–31]. The percentage of each component varied somewhat depending on the driving cycle and vehicle but remained relatively constant with the sulfate and the phosphate forms being the major (~80%) components [1].

The USEPA subsequently agreed to the use of commercially available manganese phosphate (as hureaulite), MnSO_4_, and Mn_3_O_4_ for the inhalation studies rather than rely on the use of a dynamometer system that used MMT in the fuel source to generate exhaust particulates. This decision also allowed the Hamner research team to achieve the high exposure concentrations (mg/m^3^) required to conduct the study. Such high concentrations were necessary to ensure tissue accumulation so the data could be used to inform the subsequent PBPK models. Similar aerosol concentrations and particle sizes were used in the Hamner inhalation studies to facilitate direct comparison of tissue manganese burdens among these experiments. Early studies used all three forms of manganese while later experiments relied upon the form of manganese (MnSO_4_) that produced the greatest increase in brain manganese concentrations, representing a “worse-case scenario” with regards to potential brain delivery.

Another unique feature of the MMT alternative Tier 2 test program was the use of rhesus monkeys for certain studies. A substantial literature base describing species differences in neurological responses following high-dose manganese exposure existed at the time the Alternative Tier 2 program was launched. Unlike rats, manganese-exposed monkeys develop distribution patterns for this metal within the brain that mimic those seen in heavily exposed people and develop similar neuropathology and behavioral responses [32–34]. Despite these pharmacodynamic differences in response between rats and humans, pharmacokinetic data obtained from rats remained valuable because rats and primates show similar overall pharmacokinetic responses to manganese exposure, including the induction of homeostatic control mechanisms. The rat data provided critical insights into the dose-response relationship for inhaled manganese, especially during different life stages.

Once the decision to use rhesus monkeys was reached, there was a concerted effort to maximize the data that could be obtained from this study. Study endpoints included tissue manganese concentrations following MnSO_4_ exposure [35], brain magnetic resonance imaging (MRI) studies [36], respiratory tract pathology [37], catecholamine neurochemistry [38], biomarkers of neurotoxicity and oxidative stress [39], and metabolomic biomarkers of exposure [40]. The USEPA waived the GLP requirements for certain study endpoints, including the MRI study, neurochemistry measurements, and the assessment of biomarker endpoints.

Another challenge that faced the research team was conduct of the required gestational and lactational inhalational studies. These studies required the coexposure of lactating rat dams and their pups. Inhalation developmental neurotoxicity and pharmacokinetic studies generally rely on maternal separation during exposure, resulting in pup stress that may alter development, thereby confounding study results. To overcome this potential confounding variable, Vitarella and coworkers [41] developed a unique single-animal exposure cylinder designed to house a rat dam and her litter. These investigators first tested concentrations of manganese phosphate within the exposure cylinder to verify that particle concentrations within this system were equivalent to those achieved within a stainless steel 1-m^3^ inhalation chamber. Once developed, this system was then used to support the rat lactational exposure study required by the USEPA [42].

## 5. Key Research Findings

A number of significant discoveries emerged from this testing program (Table 2). While these studies are described in detail in their individual publications, several significant findings are summarized here.

**Solubility**

The Hamner particle solubility studies showed that hureaulite and Mn_3_O_4_ are relatively insoluble in simulated lung lining fluids, while MnSO_4_ is considerably more soluble in biological fluids [52]. These studies also showed that soluble manganese forms like the sulfate are more rapidly cleared from the rat lung and delivered to the rat brain following inhalation than are insoluble manganese oxide and phosphate particles.

**Direct Olfactory to Deep Brain Transport**

One project goal was to determine whether inhaled manganese could be transported to the brain directly via the olfactory nerve. Interest in this topic was sparked by the knowledge that the olfactory system forms a direct interface between the air and the brain. The Hamner conducted studies in rats using short-term (90 min) inhalation exposure to radiolabeled (^54^Mn) aerosols in rats with one occluded nostril, thus restricting olfactory transport of manganese to only one side of the rat brain. These novel studies dramatically demonstrated that the olfactory route contributes the vast majority (>90%) of the ^54^Mn found in the olfactory pathway of the rat brain up to 8 days following acute inhalation exposure. To our knowledge, this was the first study to demonstrate that an inhaled metal could be delivered to the olfactory brain regions directly via the olfactory nerve. Although olfactory transport rapidly delivers manganese to brain structures in the olfactory pathway, it appears to be relatively slow (and inefficient) in delivering inhaled manganese to the rat striatum and other more distant brain structures [53].

**Addition of Inhaled Manganese to Existing Oral Exposures**

Individuals with either deficient or excessive manganese tissue burdens have been postulated to be at increased risk for manganese toxicity following inhalation exposure [45]. Two related 14-day inhalation studies conducted by the Hamner demonstrated that manganese body burden does not influence brain manganese concentrations following inhalation [44, 54]. These studies placed postnatal day (PND) 10 rats on specially formulated diets that contained 2, 10, or 100 ppm manganese. The lowest and highest diets were chosen in order to provide the animals with a marginally deficient or high-normal level of manganese. The 10 ppm manganese diet used in the studies met rodent dietary guidelines. Once tissue manganese concentrations stabilized (i.e., after 2 months on the special diets), rats were exposed by whole-body inhalation for 6 hr/day on 14 consecutive days to MnSO_4_ or Mn_3_O_4_ at concentrations equivalent to 0, 0.03, or 0.3 mg Mn/m^3^. Feeding the 2 ppm manganese diet was associated with a number of effects, including reduced body weight gain, decreased liver manganese concentrations, and reduced whole-body manganese clearance rates. Although rats kept on this diet and then exposed to 0.3 mg Mn/m^3^ developed increased manganese concentrations in some tissues, the studies did not demonstrate any statistically significant diet and inhalation interactions on brain manganese concentrations.

**Neonatal Exposures**

Concerns have been raised regarding increased risk of neonates for manganese-induced neurotoxicity [32]. The increased sensitivity of neonatal animals to manganese appears to be due in part to their ability to develop higher brain manganese levels than adults when faced with equivalent or lesser manganese exposures by high-dose oral gavage [51]. Factors influencing this increased susceptibility of neonatal animals may include enhanced manganese absorption from the gastrointestinal tract, an incompletely formed blood-brain barrier, and a greatly reduced basal biliary manganese excretory rate until weaning. However, information was more limited regarding the potential risks in neonates exposed to airborne manganese. To further assess this concern, the Hamner exposed rat dams and their offspring to air or MnSO_4_ (0.05, 0.5, or 1 mg Mn/m^3^) for 6 hr/day, 7 days/week starting 28 days prior to breeding and from PND 1 through 18. The experimentally determined manganese concentration in neonatal striatum and the model-predicted AUC for this brain region did not imply significantly higher exposures in the pups when compared to those in adults up to the inhaled dose of 1 mg/m^3^ [55, 56]. Despite the virtual absence of basal biliary excretion in neonatal rats, they appear to induce their biliary excretion when challenged with excess manganese through the oral route [57]. This inducible excretion in neonates was shown to be applicable to inhaled manganese as well to a level comparable to adults [56]. Because neonates have an increased requirement for manganese for optimal brain development, further elaboration of the dose-response relationship between brain manganese levels and neurotoxicity may further elucidate the potential vulnerability of neonatal brain to manganese.

**Studies in Rhesus Monkeys**

The Hamner inhalation study that exposed juvenile rhesus monkeys to MnSO_4_ is amongst the most critical completed to date with manganese. In this study, one group of monkeys was exposed to either air or MnSO_4_ (0.06, 0.3, or 1.5 mg Mn/m^3^) for 65 exposure days (6 hr/day, 5 days/week) before tissue analysis. Additional monkeys were exposed to MnSO_4_ at 1.5 mg Mn/m^3^ for 15 or 33 exposure days and evaluated immediately thereafter or for 65 exposure days followed by a 45- or 90-day delay before evaluation. Monkeys exposed to MnSO_4_ at ≥0.06 mg Mn/m^3^ developed increased manganese concentrations in the globus pallidus, putamen, olfactory epithelium, olfactory bulb, and cerebellum. Absolute manganese concentrations in the MnSO_4_-exposed monkeys demonstrated a decreasing peripheral-central concentration gradient within the olfactory system (i.e., olfactory epithelium ≫ olfactory bulb > olfactory tract > olfactory cortex). These data are consistent with direct olfactory transport of inhaled manganese. Increased pallidal manganese concentrations were evident by brain MRI and further confirmed by atomic absorption spectrometry analysis of the tissues [36]. MRI changes seen in this monkey study were similar to those reported in welders that have had high manganese exposure and subsequently developed bilateral hyperintensity on T1-weighted images in the globus pallidus and other brain regions [7]. Signal hyperintensities could not be visualized by MRI between the olfactory bulb and more distal sites, suggesting that direct translocation of manganese from the olfactory bulb to the globus pallidus did not occur in the manganese-exposed monkeys. Metabolomic analysis of serum and chemical analysis of brain tissues from the MnSO_4_-exposed monkeys revealed changes indicative of oxidative stress at higher exposure concentrations [39, 40]. Dorman and coworkers [37] also reported that exposure of monkeys to MnSO_4_ at 1.5 mg Mn/m^3^ for ≥15 exposure days resulted in increased lung manganese concentrations, mild subacute bronchiolitis, alveolar duct inflammation, and proliferation of bronchus-associated lymphoid tissue. Bronchiolitis and alveolar duct inflammatory changes were absent 45 days after exposure, suggesting that these lesions are reversible upon cessation of subchronic high-dose manganese exposure.

## 6. Lessons Learned

The work described herein represents the most extensive set of pharmacokinetic studies performed to date under the USEPA Alternative Tier 2 requirements. The pharmacokinetic data that was collected through this testing program has dramatically improved our understanding of the health risks posed by manganese. These studies have led to an improved understanding of the exposure conditions that lead to increased concentrations of the metal within the adult and developing brain and other tissues. This work has also led to the development of predictive, PBPK models for inhaled manganese that relate lung, brain, and other tissue manganese concentrations to exposure concentrations [2]. These PBPK models should lead to the development of human health risk assessments for inhaled manganese that will consider both its essentiality and neurotoxicity.

This testing program also represents an example of productive industry/government cooperation despite the challenging regulatory climate under which it commenced [22]. Evidence of cooperation was manifested by the sponsor's willingness to conduct additional studies that were outside of the scope of the required testing program. Some examples included work to evaluate olfactory transport of manganese following inhalation, short-term (2 weeks) pharmacokinetic studies, and experiments designed to examine the elimination kinetics of inhaled manganese (see Table 1). Many of these studies were needed to develop the framework for subsequent development of the PBPK models. In addition, the sponsor also voluntarily developed the human PBPK models, as only the animal models were required by USEPA. All parties involved in this program shared a common desire to develop the most robust set of experiments possible. Figure 1 shows the extensive review and oversight by EPA and others that contributed to the development of robust experimental protocols. In addition, all parties involved recognized the value of the development and use of novel technologies (e.g., inhalation exposure systems for lactating rats) and incorporation of a wide array of endpoints, including MRI. There was also great value in conducting the work at a multidisciplinary independent research institute that could help facilitate discussions between the USEPA and the research sponsor. Incorporation of additional manganese TAPs composed of external experts also built on the Hamner's rich history of independent external peer review of their research programs. This testing program also benefited from multiple postdoctoral fellows and undergraduate researchers; thus, this program also played a key role in training new scientists.

Finally, the MMT testing program is aligned with the National Research Council vision described in their report entitled *“Toxicity Testing in the 21st Century: A Vision and a Strategy”* [58]. The three main components of the NRC vision for the future of toxicity testing are chemical characterization, toxicity testing, and dose-response and extrapolation modeling. The NCR vision also describes a paradigm shift away from a focus on identifying adverse effects observed in experimental animals at high doses toward identifying and avoiding biologically significant perturbations of key toxicity pathways [59]. The identification of transition points between normal function and exposures that lead to accumulation and effects of an agent, as well as a consideration of the adaptive changes that respond to initial perturbations and function to maintain homeostasis, is a key to the NRC vision (see [58, Figure  2.2]). In our opinion, significant changes in tissue manganese concentrations represent a critical early step in the development of manganese neurotoxicity. Importantly, this work has identified a dose-dependent transition in manganese kinetics, which is a point where there is a change in the relationship of tissue accumulation as a function of dose [60, 61]. The pharmacokinetic data and PBPK modeling have shown that background tissue manganese levels are well maintained at low to moderate exposure levels, due to the existence of homeostatic control mechanisms, such as increased biliary manganese excretion, that serve to regulate tissue levels. Only once those mechanisms are overwhelmed do tissue levels start to increase significantly. Due to the existence of this dose-dependent transition, only roughly one order of magnitude separates the point of departures on which previous manganese risk assessments are based and a level of exposure at which no significant changes in Mn tissue concentration is predicted to occur in target tissues. This may indicate that large uncertainty factors are not necessary when extrapolating high-dose occupational exposure levels to low environmental exposure levels for the general population for an essential element such as Mn. The human PBPK models for Mn that emerged from this program can be used to further analyze the relationship between exposure and target tissue concentration and provide a consistent dose-response relationship for the effects of Mn regardless of exposure route and duration [43]. The models can be used to extrapolate to lower exposures to determine a concentration at which no significant effect on brain concentrations would be expected compared to normal variation. The human PBPK model can consider all life stages (fetuses, neonates, adults, and old age), both genders, pregnancy, and form of Mn. This model will be a critical tool for the quantitative risk assessment of environmental and occupational exposures to Mn. The model and these applications are discussed in more detail in Part 2 of this series [2].

## Figures and Tables

**Table 1:** Overview of the pharmacokinetic studies conducted in response to the Alternative Tier 2 test program for MMT. The following forms of manganese (Mn) were used in these studies: Mn tetroxide, Mn sulfate, and Mn phosphate. Some studies also involved intravenous administration of radiolabeled Mn chloride (^54^MnCl_2_) to assess whole-body (WB) clearance.

Pharmacokinetic endpoint of interest	Regulatory Status	Animal Species	Manganese species and exposure conditions	Primary publication(s)
Particle solubility and dissolution kinetics	Voluntary	Rat	Phosphate, sulfate, and tetraoxide—intratracheal instillation 0, 0.04, 0.08, or 0.16 *μ*g Mn/g Rats killed at 0, 1, 3, or 14 d	[42]
Exposure-response and WB clearance	Voluntary	Rat	Phosphate inhalation 6 h/d for either 5 d/wk or 7 d/wk at 0, 0.03, 0.3, or 3 mg Mn/m^3^ for up to 14 d	[43]
Particle solubility and WB clearance	Voluntary	Rat	Sulfate and tetroxide inhalation 6 h/d for 7 d/wk at 0, 0.03, 0.3, or 3 mg Mn/m^3^ for 14 d	[44]
Diet-inhalation interaction and WB clearance	Voluntary	Rat	Sulfate or tetroxide inhalation 6 h/d for 7 d/wk at 0, 0.03, or 0.3 mg Mn/m^3^ for 14 d Low (2 ppm), sufficient (10 ppm), or high (100 ppm) Mn diets	[45, 46]
Olfactory transport of Mn	Voluntary	Rat	Chloride and phosphate inhalation ~0.5 mg Mn/m^3^ for 90 min Occluded nostril model	[47, 48]
Individual susceptibility WB clearance Nasal pathology	Required	Rat	Inhalation Exposed 6 h/d for 5 d/wk to sulfate at 0.01, 0.1, or 0.5 mg Mn/m^3^ or phosphate at 0.1 mg Mn/m^3^ for up to 90 d Adult male, adult female, and senescent male	[49, 50]
Individual susceptibility	Required	Rat	Sulfate inhalation Exposed 6 h/d for 7 d/wk at 0, 0.05, 0.5, or 1 mg Mn/m^3^ throughout the majority of pregnancy or lactation.	[41, 51]
Species differences Brain imaging Respiratory tract pathology and neurochemistry	Required	Rhesus monkey	Sulfate inhalation Exposed 6 h/d for 5 d/wk at 0, 0.06, 0.3, or 1.5 mg Mn/m^3^ for up to 90 d.	[34–38]

**Table 2:** Notable scientific contributions derived from the MMT Alternative Tier 2 test program.

Pharmacokinetic endpoint	Key Finding(s)
Chemical form of manganese	Lung uptake and brain (tissue) delivery is highly influenced by solubility (sulfate ≫ phosphate > tetroxide)
Dose and duration dependences	Manganese uptake and elimination rates depend on exposure dose and exposure duration Delivery to the brain and development of pseudosteady manganese concentrations develop rapidly Biliary excretion shows similar time and concentration dependencies.
Homeostatic control	Manganese concentration in brain remains controlled at low levels of exposure and accumulates at air concentrations >10–50 *μ*g/m^3^
Dose metrics	Dose rate rather than cumulative dose appears to be the appropriate dose metric at low levels of exposure
Route of exposure	The observed pharmacokinetic differences between dietary and inhaled manganese can be attributed to rates of uptake and elimination required to achieve the same target tissue doses
Olfactory transport	Inhaled manganese is taken up by the nasal olfactory epithelium and transported directly via the olfactory nerve to the olfactory bulb
Species differences	Despite specific pharmacokinetic differences between rats and nonhuman primates, similar overall pharmacokinetic responses to and homeostatic controls of manganese were observed across species
